# SARS-CoV-2 Syncytium under the Radar: Molecular Insights of the Spike-Induced Syncytia and Potential Strategies to Limit SARS-CoV-2 Replication

**DOI:** 10.3390/jcm12186079

**Published:** 2023-09-20

**Authors:** Hashim Ali, Asma Naseem, Zaheenul Islam Siddiqui

**Affiliations:** 1 Department of Pathology, University of Cambridge, Addenbrookes Hospital, Cambridge CB2 0QQ, UK; 2 Infection, Immunity and Inflammation Research and Teaching Department, Great Ormond Street Institute of Child Health, University College London, London WC1N 1DZ, UK; 3 Diabetes and Obesity Research Center, NYU Grossman Long Island School of Medicine, New York, NY 11501, USA

**Keywords:** COVID-19, SARS-CoV-2, spike, syncytia, TMEM16F, niclosamide

## Abstract

SARS-CoV-2 infection induces non-physiological syncytia when its spike fusogenic protein on the surface of the host cells interacts with the ACE2 receptor on adjacent cells. Spike-induced syncytia are beneficial for virus replication, transmission, and immune evasion, and contribute to the progression of COVID-19. In this review, we highlight the properties of viral fusion proteins, mainly the SARS-CoV-2 spike, and the involvement of the host factors in the fusion process. We also highlight the possible use of anti-fusogenic factors as an antiviral for the development of therapeutics against newly emerging SARS-CoV-2 variants and how the fusogenic property of the spike could be exploited for biomedical applications.

## 1. Introduction

Cell–cell fusion events occur in many physiological processes, including fertilisation, development, immunity, and astrocytes, which allow the sharing of cellular contents, forming functional syncytia [1,2,3]. However, the fusion of cells which do not fuse naturally leads to several human diseases, including preeclampsia, myopathies, male infertility, cancer, and neurodegeneration [4,5,6,7]. Although the formation of pathological syncytia by several human viruses has been reported earlier [8,9], it gained massive attention when several groups noticed the abnormal fusion of cells in the biopsy of lungs from patients who died with COVID-19 [10,11]. It is widely accepted that SARS-CoV-2 is associated with the destruction of the lung epithelium, immune dysfunction, thrombosis, and thrombocytopenia [10,12,13,14,15,16]. A similar phenotype is seen in HIV-1 infection, where it overwhelms the host immunity by either inducing lymphopenia or initiating a senescence-mediated inflammatory response [17,18]. In addition to extensive tissue damage, pathogenic viruses exploit the syncytium for their productive infection because it protects viruses from host immune sensing mechanisms [19]. However, cell fusion events also generate micronuclei which are sensed by host innate immune sensors during SARS-CoV-2 and measles virus infection [20,21]. This supports the hypothesis that fused pneumocytes or other cells in the lungs of COVID-19 patients accelerate the production of cytokines, like IFNs and TNFa, thus worsening the disease severity. Of note, several reports also suggest that SARS-CoV-2 directly infects cardiac cells and induces myocarditis [22,23,24], and the spike-mediated fusion of cardiac cells might be associated with arrhythmic risk in COVID-19 patients [25,26]. Heart cells, particularly mature cardiomyocytes, harbour the most complex intracellular structure called the sarcomere, which is difficult to remodel [27]. Importantly, the mechanism by which the SARS-CoV-2 spike copes with the cardiac cytoskeleton to trigger the fusion of cardiac cells (contractile cells) requires further investigation.

SARS-CoV-2 is an enveloped, positive-sense single-stranded RNA (+ssRNA) virus belonging to the beta-coronavirus genera that also includes SARS-CoV and MERS-CoV (Middle East respiratory syndrome coronavirus), which are also responsible for prior outbreaks in the years 2003 and 2012, respectively [28,29,30,31]. These viruses take advantage of spike-surface glycoproteins to enter host cells by binding to the cognate receptors. SARS-CoV-2 and SARS-CoV primarily rely on the ACE2 receptor for their entry into the host cells, while MERS-CoV engages with the DPP4 (transmembrane dipeptidyl peptidase 4) for its entry [32,33].

**Figure 1:**
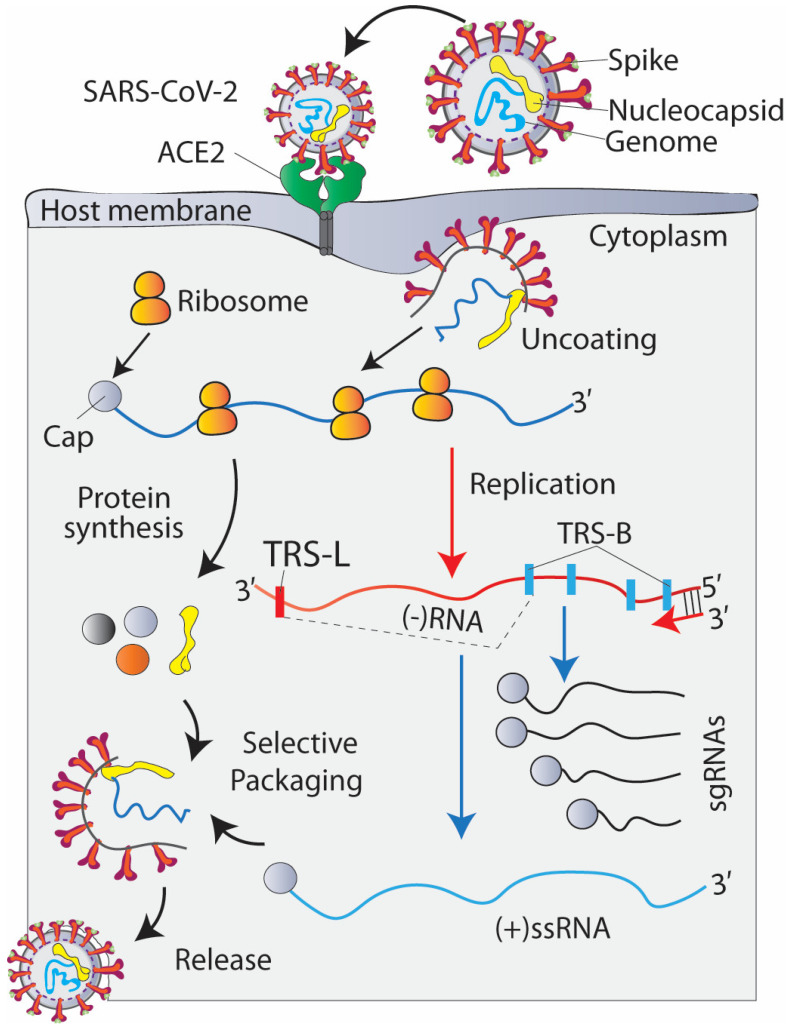
Illustration of SARS-CoV-2 virion structure and replication cycle. The SARS-CoV-2 virion structural protein spike and nucleocapsid are shown in red and yellow colour, respectively, while the genome is shown in blue colour. The positive-sense, single-stranded RNA genome (+ssRNA) is encapsidated in the protein shell. Upon virus entry, the viral genome is released into the cytoplasm. The viral +ssRNA is directly translated by host translational machinery, followed by the synthesis of full-length negative-sense RNA copies for replication and sub-genomic negative-sense RNAs (−sgRNA) to make the positive-sense sub-genomic RNAs (+sgRNAs). The negative-sense RNA synthesis requires a template switch from a body transcription regulatory sequence (TRS-B) to the leader TRS (TRS-L) to generate sub-genomic mRNA. Template switching can take place at any TRS-B in viral genome and co-operatively result in the production of other sub-genomic mRNA species. which acts as templates for the protein synthesis. which is required for viral replication and assembly of viral particles.

During the SARS-CoV-2 cell entry, upon binding with the ACE2 receptor, the spike undergoes proteolytic priming, followed by conformational changes, which initiate the fusion of the viral membrane with the host cell membrane and the transfer of its genome into the cytoplasm (Figure 1).

Like other +ssRNA viruses, SARS-CoV-2 also exploits a number of host factors to rapidly translate its genome to form a functional replication complex [34], which is required for genome replication and packaging, and the release of progeny virions.

The spike is a class I fusion protein; once expressed on the surface of the infected cells, it interacts with the ACE2 receptor of neighbouring cells and triggers the cell–cell fusion process, leading to large muti-nucleated syncytia. Comparatively, the SARS-CoV spike is less fusogenic than that of SARS-CoV-2 and MERS-CoV, possibly because of the absence of a furin cleavage site at the S1/S2 boundary [12,35].

This review aims to provide an overview of the virally encoded fusion proteins, focusing on the SARS-CoV-2 spike protein and the host factors modulating its fusogenicity. Secondly, we highlight the possible use of anti-fusogenic factors as antivirals for the development of therapeutics against the emerging novel SARS-CoV-2 variants.

## 2. Characteristics of the Virus-Encoded Fusion Proteins

**Figure 2:**
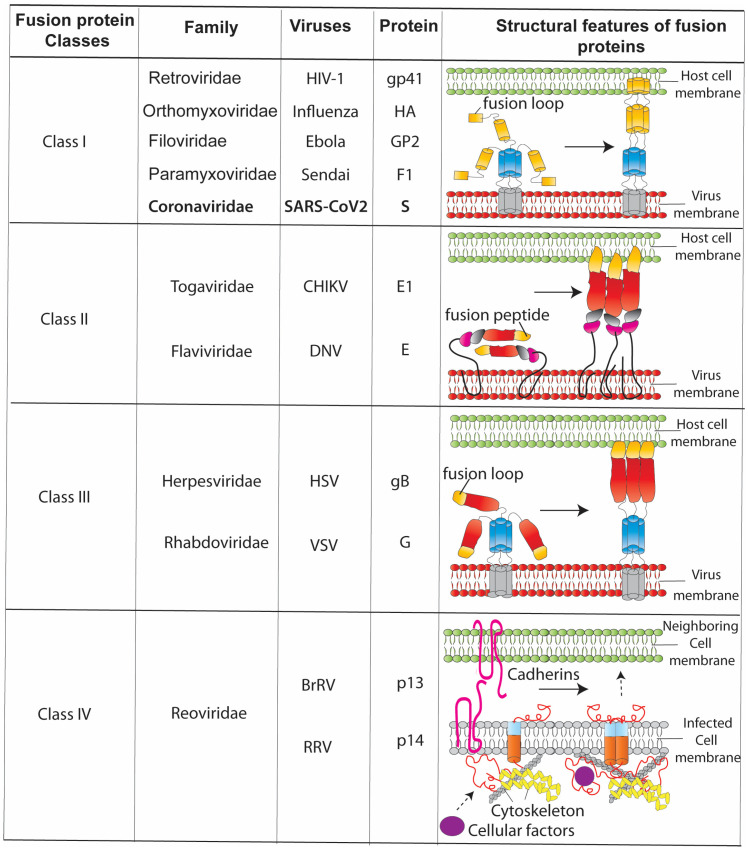
List and structural features of class I to IV fusion proteins. Class I fusion proteins (HA, gp41, and envelope glycoprotein) contain alpha-helical structures with proteolytically primed fusion peptides at the amino-terminal ends. Class II fusion proteins (e.g., envelope protein E, VSV-G, and gB) contain β-sheets arranged in a three-domain fashion (DI, DII, and DIII), and domain II contains a folded fusion loop. In contrast, class III fusion proteins share the structural features of class I and II fusion proteins (contain the α-helical domain and β-sheet domains with fusion loops). Class IV fusion proteins are non-structural, single-pass transmembrane proteins, called FAST (fusion-associated small transmembrane), expressed in infected cells after viral genome translation. These proteins are exclusively present in the orthoreovirus and aquareovirus genera of the reoviridae family. (HIV-1: Human immunodeficiency virus 1, CHIKV: Chikungunya virus, DNV: Dengue virus, HSV: Herpes simplex virus, VSV: Vesicular stomatitis virus, BrRV: Broome orthoreovirus, and RRV: Reptilian orthoreovirus).

To productively replicate, viruses require full access to the intracellular resources of the host cell. Viruses enter the cell through the direct interaction of the viruses’ surface proteins with the cognate receptors on the target cells. Depending on the virus, this specific interaction may result in receptor-mediated endocytosis. Primarily, enveloped viruses express their fusogenic glycoproteins on their surface, which bind to receptors and assist in entering the host cells by the fusion of the viral membrane with the cell membrane. The viral fusion proteins can be grouped into four classes based on their types and fusion mechanisms (Figure 2).

Detailed molecular properties and fusion mechanisms associated with different viral proteins are well-documented and reviewed [36,37,38,39]. The first three classes of fusion proteins (class I–III) are large glycoproteins that help the viruses to enter cells [40].

Class I fusion proteins are alpha-helical in structure and have proteolytically primed fusion peptides at their amino-terminal ends that tie the virus to the host cell plasma membrane [41]. Moreover, conformational changes in class I fusion proteins can be pH-dependent (e.g., HA and influenza virus), pH-independent (e.g., gp41 and HIV-1), or both (e.g., envelope glycoprotein and avian alpha-retroviruses).

Class II fusion proteins are composed of β-sheets arranged in a three-domain fashion (DI, DII, and DIII), of which domain II contains a folded fusion loop similar to the class I fusion proteins. Conformational changes in class II fusion proteins are mainly triggered by pH variations (e.g., E envelope protein and flaviviruses). In contrast, class III fusion proteins share the structural features of class I and II fusion proteins (containing both the α-helical domain and β-sheet domains with fusion loops). These proteins are expressed on the surface of the vesicular stomatitis virus (VSV-G protein) and herpes viruses (gB and membrane glycoprotein B).

The most common characteristics of classes I, II, and III fusion proteins are that they all harbour large ectodomains (which can extend >10 nm from the plasma membrane), form functional trimers during membrane fusion, and fold into stable trimers in the post-fusion conformation. The large ectodomains of the fusion proteins allow them to easily reach the adjacent cells (bypass the inter-cell membranes gaps and hindrance because of cell surface molecules) and anchor to the opposing membranes [42,43]. Once the fusion proteins anchor the viral and host cell membranes, conformational changes initiate the fusion protein to fold back, pulling the membranes into close contact and forming a stable fusion structure that promotes fusion [42,44,45,46].

Class IV fusion proteins are non-structural, single-pass transmembrane proteins which are not present on the surface of the viruses but get expressed in infected cells after viral genome translation [47]. These fusion proteins are exclusively present in the orthoreovirus and aquareovirus genera of the reoviridae family [48]. Reovirus fusion proteins are called FAST (fusion-associated small transmembrane) proteins, which are exported to the plasma membrane of the infected cells and induces cell–cell fusion [48]. Comparatively, FAST proteins are small (100–200 amino acids in length) with three functional domains: a short ectodomain (N-terminal), a single transmembrane domain, and a large cytoplasmic tail, all of which are essential for driving the fusion process [38]. Unlike enveloped virus fusion proteins, FAST proteins do not bind to specific cellular receptors [49,50], but exploit the intracellular machinery to initiate the fusion and formation of a large multinucleated syncytium [51]. Owing to their short ectodomain, FAST proteins are unable to cope with inter-cell repulsive forces [50,52,53]. Recently, it has been proposed that FAST proteins bypass the inter-cell barrier by exploiting the host actin cytoskeleton and adheren complexes [51,54,55].

## 3. Molecular Properties of SARS-CoV-2 Spike

**Figure 3:**
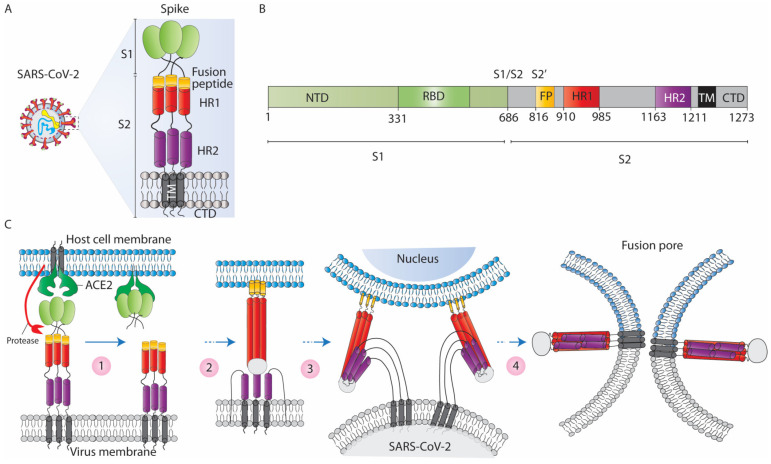
Schematic of the SARS-CoV-2 virus and spike-induced fusion mechanism. (**A**) Architecture view of the spike fusion protein; in a trimeric spike, the S1 forms a globular head essential for receptor binding, while S2 forms a narrow stalk, fusion peptide (FP), and two heptad repeat regions (HR1 and HR2) and transmembrane domain (TM) which helps spike protein to anchor viral and cell membranes. The S2 initiates membrane fusion process. (**B**) An overview of the functional domains of the S protein. N-terminal domain (NTD), receptor-binding domain (RBD), fusion peptide (FP), heptapeptide repeat regions (HR1 and HR2), transmembrane anchor (TM), and C-terminal domain (CTD). (**C**) Schematic of spike-induced fusion process: (1) spike protein primed by cellular proteases at the S1/S2 region releases the S1 domain, which allows HR1 to extend and thrust the FP into the target cell plasma membrane. (2) The FP tie the target membrane while the TM domain anchors the fusion machinery to the viral or cell membrane. (3) Interactions between HR1 and HR2 follow in a hairpin-like foldback, conquering the energetic barrier to fusion and bringing the opposing membranes to proximity. (4) A hemi-fusion step followed by the association of HR1 and HR2 and the corresponding membrane fusion results in the formation of a fusion pore that will progressively and finally merge the viral membrane or cytoplasm of cells.

The spike protein of SARS-CoV-2 is a ~180 kDa surface glycoprotein with a large extracellular N-terminus, a single transmembrane (TM) domain, and a small cytoplasmic C-terminal tail (Figure 3B). On the surface of the virion, a spike exists in trimeric form, which decorates virions with crown-like structures (Figure 3A). The spike is synthesised in an inactive form like other class I fusion proteins. For activation, it is highly dependent on the host proteases [35,56,57,58]. A major proportion of the spike produced in the infected cell is trafficked via the ER and undergoes glycosylation in the Golgi to assemble and form new viral progeny. However, a smaller fraction of the spike escapes from the ER/Golgi and traffics to the plasma membrane [59], where it interacts with the ACE2 molecules of neighbouring cells and triggers cell–cell fusion.

The extracellular N-terminus of the spike consists of two large subunits, S1 and S2. The S1 subunit contains the receptor-binding domain (RBD) and is essential for virus attachment to the target cells. The high binding affinity of RBD to the ACE2 receptor [60] makes it a perfect target for neutralising antibodies [61,62]. Any modification or mutation in the RBD critically affects the ACE2-spike association. The S2 subunit includes a fusion peptide (FP), two heptapeptide repeat domains (HR1 and HR2), a transmembrane domain, and the small C-terminal cytoplasmic tail. The S2 subunit is essential for viral entry by inducing the fusion of the virus to the host cell membrane [63,64,65]. Mechanistically, FP links the host membrane to the fusion assembly complex (Figure 3C), while the assembled trimeric forms of HR1 and HR2 constitute a 6-helical bundle (6-HB) which draws the linked membranes into a proximal position required for the class I fusion proteins [66].

Like other enveloped viral fusion proteins, the spike easily overcomes the repulsive barriers that hold the membranes apart. Once the energetic fusion barrier is reduced, the outer leaflets of the membranes fuse and form the intermediate hemi-fusion state [66]. The hemi-fusion state of the coupled membranes is followed by the post-fusion hairpin conformation, which leads to the formation of an orifice that extends to form a single membrane. This allows the virus/fusion proteins to access the host cytoplasmic components or mixing of the cytoplasmic contents of the homo/heterogeneous cells.

The highly dynamic and controlled nature of the virus-induced fusion process makes it an attractive target for developing broad-spectrum antivirals. For instance, a peptide derived from the envelope protein of the Zika virus not only inhibits the Zika virus infection but is also effective against other flaviviruses [67]. Since the spike HR1 and HR2 domains are relatively conserved in coronaviruses [68], the HR regions of the S2 domain are a promising target for developing fusion inhibitors against SARS-CoV-2 infection.

## 4. SARS-CoV-2 Spike Fusogenicity

**Figure 4:**
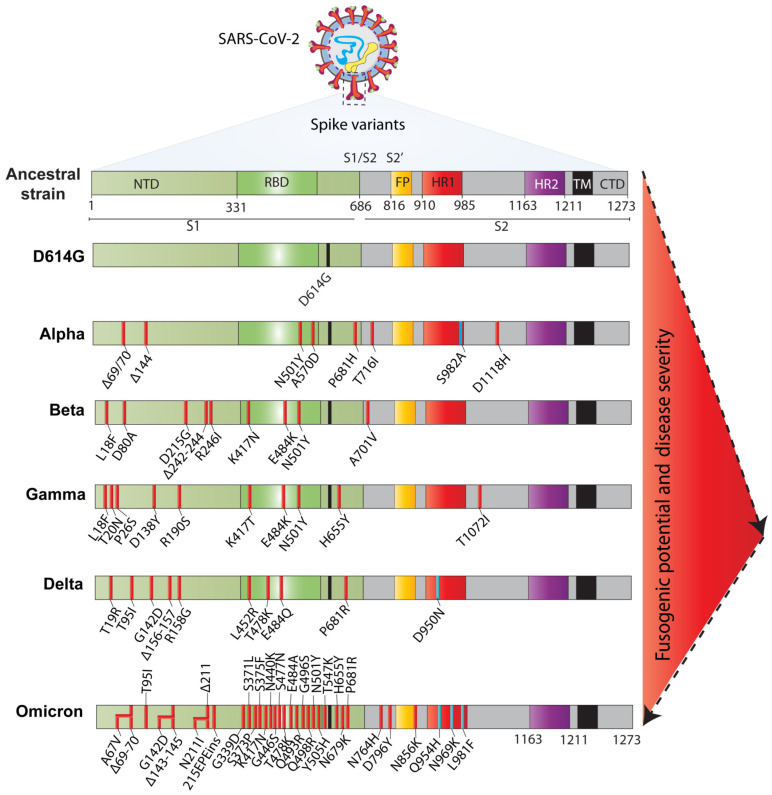
Schematics of emerging SARS-CoV-2 variants and spike’s fusogenicity. The ancestral SARS-CoV-2 strain isolated in Wuhan in 2019 has evolved into many variants, often with numerous spike mutations. Most spike variants (D614G, alpha, beta, gamma, and delta) show higher fusion potential than the ancestral strain, except omicron. The red triangle indicates the fusogenic potential of the spike variants.

Despite having the proofreading potential of viral RNA polymerase, the SARS-CoV-2 genome is rapidly evolving and persistently accruing mutations, many of which also happen in the spike-coding sequence [69,70]. Mutations in the spike may significantly impact the protease-mediated priming, stability, affinity towards receptors, fusion efficiency, and resistance to vaccine-induced immunity. Because of the highly immunogenic nature of the spike, and the associated evolutionary pressure during propagation among the infected human population, the ancestral strain was rapidly replaced with newly emerging variants. After the outbreak of SARS-CoV-2 in December 2019, the first variant, D614G, which harboured a mutation in the S1 domain of the spike, emerged and became the most dominant strain in Europe and spread to other continents [71]. It has been shown that the spike variants possessing the D614G mutation are rapidly processed by the host furin protease. This modification increases the virus infectivity and transmission rate [72]. Interestingly, the spike containing the D614G mutation also shows higher cell–cell fusion compared to the ancestral strain, possibly due to its higher affinity towards the ACE2 receptor and enhanced furin protease-mediated processing [72]. Due to the continuous evolutionary pressure, SARS-CoV-2 continued to mutate its genome to optimise its replication in the vaccinated and unvaccinated populations. Of note, the accumulation of several mutations in the viral genome is responsible for emerging novel SARS-CoV-2 variants called “variants of concern (VOCs)”. To address this concern, the World Health Organization (WHO) classified five SARS-CoV-2 variants of concern—alpha (B.1.1.7), beta (B.1.351), gamma (P.1), delta (B.1.617.2), and omicron (B.1.1.529). These VOCs also harbour critical mutations in the spike-coding sequence (Figure 4), some of which are more infectious than others, and also allow the virus to evade vaccine-induced antibodies [73]. However, their associated pathologies are still unclear and require further investigation.

To date, many research groups have systematically compared the infectivity and fusogenic potential of the spike of the VOCs and found that most of them, except omicron, form rapidly and relatively larger syncytia in different types of cells [74,75]. Both alpha and beta variants show similar replication kinetics in primary airway cells and several cell lines, and display enhanced syncytia formation compared to the ancestral strain [74,75]. This suggests that newly emerging pathogenic variants also show high fusogenicity, thus underlining the disease severity associated with the spike-induced syncytia. At the end of 2020, the delta variant appeared in India and was responsible for the massive number of hospitalisations and the gigantic COVID-19 wave [76]. In a report, Saito et al. showed that the delta variant is highly pathogenic and rapidly deteriorates lung tissues and function, possibly because of the extensive fusogenicity it demonstrates in the Syrian hamster animal model [77]. Moreover, the substitution of proline to arginine at the 681 residue (P681R) in the spike of the delta variant is proposed to drive the higher viral pathogenicity and fusogenicity, and its resistance for the neutralising antibodies [77,78,79]. Structurally, the proline 681 residue is proximal to the furin protease cleavage and o-glycosylation sites [80,81]. Therefore, arginine substitution abolishes o-glycosylation, and, subsequently, fastens spike processing by furin protease, eventually making it highly contagious and fusogenic [82]. The switching of proline to arginine at the 681 positions also appeared in the alpha variant, suggesting that it regulates the post-translational modification of the spike and modulates the host factors for a higher pathogenicity and fusogenicity.

A super-spreader variant called omicron was recently identified, shortly becoming the most dominant variant worldwide. Sequencing data suggested that it harbours >97 mutations across the genome, with 33 nonsynonymous or indel mutations in the spike-coding region [83,84]. Suzuki et al., while comparing the fusion efficiency, noticed that the omicron variant shows a relatively higher expression on the plasma membrane than the delta variant, while being lower than D614G. However, omicron is less fusogenic in cell-culture-based fusion assays than the delta and other D614G early-pandemic variants [75]. Finally, over time, the spike accumulated a large number of mutations which, subsequently, impaired its fusogenic capacity, which is directly correlated with the pathogenicity and severity of the omicron variant. This reduced fusogenicity of the omicron variant might be because of its inefficient cleavage by the host proteases, or the mutations in the HR1 domain might impair the interaction with HR2, which, subsequently, reduced the membrane-anchoring property of the fusion peptide.

The small C-terminal cytoplasmic domain of the spike contains essential signature motifs that interact with several host factors during the assembly and budding of viral progeny [59]. A dibasic ER-retrieval (2719-KLHxx-1273) signal sequence at the C-terminus allows spike recycling via the binding to the host coatomer complex I (COPI) and exit from the Golgi compartment [85]. Moreover, the second di-acidic ER-export signal sequence (1257-DEDDSE-1262) binds with COPII, which directs its efficient exit from the endoplasmic reticulum. A small fraction of spike leakage from the ER–Golgi allows its cytosolic and cell surface distribution, remodeling the host signalling and potentially disrupting the calcium signalling and chloride current, which, subsequently, activates the TMEM16F (more details in Section 5). The deletion of the last ~20 amino acids at the C-terminus permits the spike to easily travel to the plasma membrane in the pre-activated state, which induces rapid and large syncytia formation [86,87].

## 5. Host Factors Modulating Spike-Mediated Syncytia Formation

**Figure 5:**
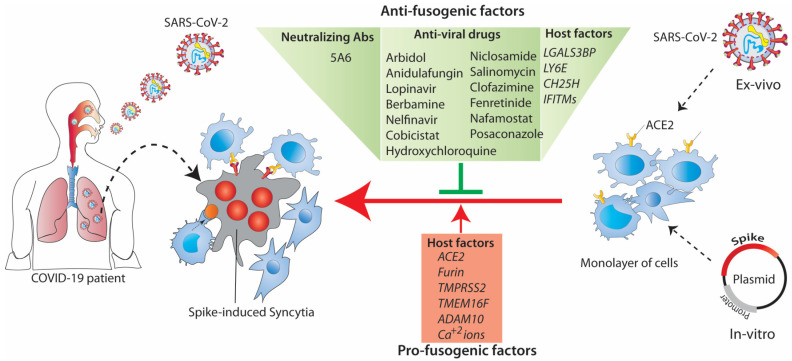
An overview of host factors regulating spike-induced syncytia formation and strategy targeting for therapeutic applications. Several FDA-approved drugs/small molecules selectively inhibit SARS-CoV-2-mediated syncytia formation. Different classes of host factors (ACE2, TMEM16F, furin, ADAM10, and calcium ions) are exploited by a spike to initiate the expansion of syncytia during infection.

As for any virus, SARS-CoV-2 extensively leans on diverse cellular factors for productive replication. The spike interacts with several host factors ranging from plasma membrane bound to intracellular organelles at the different stages of the virus life cycle. Several cellular proteases (TMPRSS2, furin, cathepsins, and ADAM) interacted with the spike, primed it at various locations, and played a crucial role in cellular tropism and the pathogenesis of SARS-CoV-2 [35,88]. In the following sections, we detail the functions of host factors and how these molecules modulate spike-induced syncytia formation (Figure 5).

ACE2 is the first host factor on the cell surface which interacts with the spike and provides a docking site on the host cells. This specific interaction is followed by virus entry into the cells [32,89,90]. ACE2/spike interaction is essential for the fusion of virus–cell membranes and plays a critical role in cellular tropism, viral transmission, and cell–cell fusion. The siRNA-mediated depletion of ACE2 molecules inhibits spike-induced cell–cell fusion and SARS-CoV-2 replication [91,92], which explains the crucial role of the ACE2 receptor virus infection.

Furin is a cellular endopeptidase that cleaves diverse pro-protein substrates ranging from receptors, to growth factors, to extracellular matrix proteins, in order to regulate physiological functions [93]. However, it also activates several proteins encoded by pathogenic viruses, such as retroviruses, herpesviruses, flaviviruses, and ortho- and paramyxoviruses [94,95,96]. Interestingly, the furin cleavage site is also found in the spike of the human coronaviruses at the S1/S2 boundary but is absent in SARS-CoV [97]. It has been assumed that the furin cleavage site insertion is crucial for the SARS-CoV-2 zoonotic transmission [98]. Secondly, the presence of furin cleavage sites in the MERS-CoV and SARS-CoV-2 spike proteins makes them highly fusogenic and is often associated with the severity of the disease [35]. Of note, the furin cleavage site is also an important determinant of SARS-CoV-2 transmission in the animal model [91]. Moreover, the furin cleavage site is also present in the spike of swine acute diarrhoea syndrome coronavirus (SADS-CoV) and is critical for syncytia formation [99]. The dependency of fusogenic viruses on the furin-mediated activation of their surface glycoproteins for virus entry and membrane fusion makes furin a potential target for antiviral development. The inhibition of furin activity could prevent the activation of viral fusion proteins and, thereby, block virus entry and syncytia formation. Furthermore, the pharmacological or genetic ablation of furin protease inhibits spike-induced syncytia formation [91]. Interestingly, inserting the furin cleavage in the SARS-CoV spike at the S1/S2 region significantly enhanced its fusogenic potential without changing its virulence [35,98,100]. Further, the modifications which altered furin-mediated spike cleavage, such as *O*-glycosylation, significantly decreased the syncytia formation [82].

Interferon-induced transmembrane proteins (IFITMs) restrict the infection of several RNA and DNA viruses by inhibiting the fusion of the virus enveloped to the host cell membrane at the hemi-fusion or pore formation stages [101,102]. However, the exact mechanisms of how IFITMs inhibit virus entry need investigation. Out of the five IFITMs encoded by the human genome IFITMs—one to three are known to be interferon-inducible [103,104]. IFITM1 predominantly localises at the plasma membrane, whereas IFITM2/3 are found in the membranes of the intracellular compartments such as endosomes and lysosomes [105,106]. These proteins also restrict the infection of human coronaviruses such as MERS-CoV, SARS-CoV, hCoV-NL63, and hCoV-229E [102,107]; however, hCoV-OC43 exploits IFITM2 and 3 to enhance infectivity [108]. Interestingly, point mutations in the structural motif of IFITM1 and 3 not only abolish the inhibitory effects on the MERS-CoV and SARS-CoV but also become proviral factors [109]. Recently, Shi and colleagues discovered that IFITMs could also inhibit SARS-CoV-2 infection by altering the membrane rigidity via their amphipathic helix. This inhibitory function is reverted in the presence of the serine protease TMPRSS2 [110]. The antiviral properties of IFITMs are potentially associated with their role in altering the membrane composition and rigidity that prevent viruses from crossing the host plasma membrane and releasing their genomes into the cytoplasm [111,112,113]. Buchrieser and co-workers showed that all IFITM members could block the spike-mediated cell fusion, but the TMPRSS2 presence abolished the IFITM’s effects [114].

ZMPSTE24 (Zinc metallopeptidase STE24) is a transmembrane metalloprotease identified as an interacting partner of IFITMs [115,116]. Like the IFITMs, ZMPSTE24 inhibits the infection of several enveloped viruses [115]. Recently, it has been shown that ZMPSTE24 protects the host against SARS-CoV-2 infection and syncytia formation [117]. However, the exact molecular mechanism of how ZMPSTE24 inhibits the replication of enveloped viruses is not precise yet.

TMPRSS2 (Transmembrane Protease Serine 2) is a host serine protease that plays a crucial role in the infectivity of certain viruses, including SARS-CoV-2 and MERS-CoV. It primes the spike and enhances virus infectivity, but it also induces structural changes in the membrane-associated receptor, which, subsequently, alters the fusion processes. Nafamostat is a drug that acts as a pharmacological inhibitor of TMPRSS2. By inhibiting TMPRSS2, Nafamostat effectively prevents the priming of the spike protein. As a result, the virus’s ability to infect host cells and cause cell–cell fusion is significantly inhibited. Therefore, Nafamostat shows promise as a potential therapeutic agent against SARS-CoV-2 and MERS-CoV infections by targeting TMPRSS2 and blocking the fusion processes required for viral entry and spread [118,119].

ADAM proteases, the shedding proteases, were recently shown to play roles in SARS-CoV-2 infection. Similar to TMPRSS2, ADAM10 and ADAM17 are membrane-bound proteases which proteolytically shed the ectodomain of diverse cell surface proteins and regulate the physiological events associated with their release [120], including the release of ACE2 and NPR1 surface molecules, which play crucial roles in SARS-CoV-2 infection [121,122,123]. Sheddase activity of the ADAMs cleaves the receptor molecules of different viruses, including enteroviruses, adenoviruses [124,125], and SARS-CoV-2 [121,122]. Recently, Jocher and co-workers have identified the function of ADAM10 and ADAM17 during SARS-CoV-2 infection using pharmacological and genetic approaches [126], showing that ADAM17 is required during the early stages of infection. ADAM10 was shown to assist in spike-mediated syncytia formation. ADAM17 also plays a crucial role in the shedding of the TNFa cytokine, which is associated with the severity of the disease and the viral load in COVID-19 patients [127,128]. DPC-333 and apratastat are enzymatic inhibitors of ADAM17, which block the shedding of TNFa [129,130]. Thus, both the ADAM10 and ADAM17 proteases are important antiviral targets against SARS-CoV-2.

Cholesterol 25-hydroxylase (CH25H) is an interferon-sensitive host enzyme that converts cholesterol into 25-hydroxycholesterol (25HC), a soluble antiviral factor [131,132]. The CH25H catalyses the oxidation of cholesterol to 25-hydroxycholesterol (25HC) in the ER and, subsequently, regulates the cholesterol level in cell membranes [133]. Interestingly, the induced expression of CH25H or the treatment of cells with 25HC inhibits the infection of various enveloped viruses by blocking the fusion of the virus to the host cell membrane [131]. Like IFITMs, CH25H also alter the membrane rigidity and curvature by employing different mechanisms. This is because CH25H can inhibit the entry of Murine gammaherpesvirus-68 (MHV-68), while IFITM3 was found to be ineffective for this activity [134]. Interestingly, CH25H/25HC not only inhibits SARS-CoV-2 infection but also tapers spike-induced membrane fusion by modulating the properties of the host cell membrane [135,136].

Lymphocyte antigen 6E (LY6E) is an interferon-inducible glycosylphosphatidylinositol-anchored host factor that is identified as an entry inhibitor of the viruses VSV and MHV-68 [134]. The cellular overexpression of LY6E effectively blocks infection by different coronaviruses, including SARS-CoV-2, by selectively targeting the ACE2-spike-mediated fusion of the virus–host cell membrane and syncytia formation [137,138].

TMEM16F is a Ca^2+^-activated chloride channel and scramblase [139]. A large-scale FDA-approved Vero cell-based drug screening was performed to identify anti-fusogenic inhibitors of the SARS-CoV-2 spike [12]. Several drugs, including Niclosamide, Clofazimine, and Salinomycin, were identified, that block spike-mediated syncytia formation. Among these drugs, the most potent was Niclosamide [12]. Niclosamide is an FDA-approved anthelminthic drug effective against SARS-CoV, MERS-CoV, Zika Virus, HCV, and human adenovirus, indicating its potential as an antiviral agent [140]. Niclosamide impairs SARS-CoV-2 infection by blocking spike-induced cell–cell fusion. Niclosamide blocks the pathological syncytia formation by blocking the cellular functions of TMEM16F. Scramblase activity of the TMEM16F catalyses the transfer of phosphatidylserine (PS), a phospholipid, from inner leaflets to the extracellular face of the plasma membrane [3,139,141]. The PS exposure to the plasma membrane signals cell–cell fusion during the physiological process, but viruses also exploit it to enter cells [142,143]. Interestingly, many viruses, including HIV-1 [144,145], Ebola [146], and alpha-herpesvirus [147], either trigger the exposure of PS on the target cell plasma membrane or carry PS on the surface to establish an early stage of infection. Mechanistically, the spike in the host cell activates the TMEM16F scramblase function, which leads to the exposure of PS on their plasma membrane, potentially initiating the cell–cell fusion in an ACE2-dependent manner [12]. During SARS-CoV-2 infection, a large proportion of the spike expressed inside the cell is present in ER–Golgi compartments, but a small proportion of it translocates to the cell surface by interacting with COPII vesicles [59]. In brief, spike protein from both locations contributes to triggering the fusion progression; ER fraction elevates the cytosolic Ca^2+^ ions level, which, subsequently, activates the TMEM16F activities, while the cell surface spike interacts with ACE2 receptor on neighbouring cells and promotes the fusion process by anchoring neighbouring cells. To fully understand the role of TMEM16F and other host factors in SARS-CoV-2 infection, syncytia formation and the development of COVID-19 requires further investigation.

Galectin-3-binding protein (Gal-3BP), a secreted glycoprotein [148], is involved in cell–cell and cell–extracellular matrix signalling [149]. Epidemiological and clinical reports suggest that patients with pathological conditions such as cancer and autoimmune diseases show the overexpression of Gal-3BP [150,151,152]. High levels of Gal-3BP were also noticed upon infection with other human viruses, including hepatitis B virus, hepatitis C virus, hantavirus, dengue virus, and HIV-1 [153,154,155,156]. It has been shown that elevated levels of Gal-3BP are associated with antiviral functions via interactions with diverse intracellular and extracellular binding partners, and the production of interferons and pro-inflammatory cytokines [157,158]. Interestingly, during the SARS-CoV-2 pandemic, several researchers identified that Gal-3BP levels increased in the circulation of COVID-19 patients, correlated with disease severity [133,159,160,161,162]. High levels of IL-6 inflammatory cytokines in COVID-19 patients [159] might be a result of the interaction of Gal-3BP with galactin-3, which induces the activation of the immune system. A detailed proteomic analysis of COVID-19 patient serum samples identified several components of the complement system and galectin-3-binding protein as binding partners of the spike protein [163]. In vitro, the overexpression of LGALS3BP cDNA, which encodes Gal-3BP in Vero cells, effectively impaired cell–cell fusion and spike-pseudoparticle entry. However, cell culture media from LGALS3BP-expressing cells do not impair spike-pseudoparticle entry, suggesting an antiviral effect of Gal-3BP on SARS-CoV-2 involving intracellular mechanisms, or its anti-viral property requires modification which is not happening in this tested model [163]. Therefore, the detailed function of the Gal-3BP in SARS-CoV-2 infection and its role in disease severity still needs further investigation.

Lipid composition in the cellular membrane is essential for its fluidity and curvature, and, thus, plays a critical role in the entry and replication of most enveloped viruses, including coronaviruses [164,165,166]. Altering the sphingolipid dynamic shows a profound effect on viral replication; for example, the glucosylceramide synthase inhibitor restricts the infection of coronaviruses and other RNA viruses [167,168]. In the case of HIV-1, a higher content of cell membrane sphingomyelin promotes HIV-1 infection by accelerating gp40-mediated membrane fusion [169]. Fenretinide, a selective inhibitor of dihydroceramide D4-desaturase 1 enzyme (DES1), is required to synthesise ceramide [170]. Interestingly, fenretinide treatment strongly inhibits spike-mediated cell fusion by decreasing membrane fluidity [171].

The calcium ion concentration is essential for cellular homeostasis and fusion events [172,173]. Ion channels and ATPase strictly regulate the cytosolic and organelle calcium ion levels. Therefore, many viruses alter cellular Ca^2+^ dynamics to form replication-competent ecosystems [174,175,176]. Several neurotropic viruses, including HIV-1 and HSV-1, alter Ca^2+^ homeostasis and are associated with synaptic loss, protein misfolding, and the damage of neurons (reviewed in [177]). Ca^2+^ ions also play a crucial role in the replication cycle of the Ebola and Rubella viruses [178,179,180], and inhibiting the calcium ion channel restricts Ebola virus entry into the host cells [181]. Like many other enveloped viruses, coronaviruses also exploit cellular calcium ions to enter the host cells [182,183]. To rapidly identify the drugs that block SARS-CoV-2 infection, Straus et al. screened a panel of drugs that block calcium channels. The study demonstrated that all the tested drugs showed inhibition, but felodipine and nifedipine effectively restricted SARS-CoV-2 entry and infection in epithelial lung cells [184]. Braga and Ali et al. observed that the SARS-CoV-2 spike amplifies the transient increase in Ca^2+^ levels, and the treatment of cells with the sarcoendoplasmic reticulum Ca^2+^ ATPase (SERCA) non-competitive inhibitors (thapsigargin or cyclopiazonic acid) not only abolishes spike-induced oscillations but also blocks the expansion of syncytia [12]. A similar effect was observed by niclosamide and clofazimine, which markedly blunted the amplitude and frequency of Ca^2+^ oscillations in cells expressing the SARS-CoV-2 spike protein. It has been postulated that Ca^2+^ directly binds to the spike fusion peptide, which, subsequently, assists in the fusion of the virus to the plasma membrane of the target cell [185]. Therefore, altering Ca^2+^ dynamics can modulate SARS-CoV-2 infection and syncytia formation.

## 6. Therapeutic Applications Targeting Spike-Mediated Cell Fusion

To tackle the resistance problem of the newly emerging variants against neutralising antibodies, several fusion inhibitors were designed to increase the armament of COVID-19 therapeutics. The HR1 and HR2 domains of the S2 subunit form a 6-helical bundle, which is fundamental to the fusion pore formation during the progression of the virus–host membrane fusion process. The highly structured and conserved features of the HR1 and HR2 domains make them a perfect target for developing viral fusion/entry inhibitors [186,187,188]. Interestingly, a helical peptide (5-helix) is designed using multiple copies of the HR1 and HR2 domains [189]. The cellular expression of the 5-helix effectively inhibits the spike-mediated syncytia formation, potentially by interacting with the HR2 of the 6-helix and blocking the formation of the fusion pore. Recently, an HR2 sequence-based lipopeptide fusion inhibitor called IPB02 showed potent activities in inhibiting spike-mediated cell fusion and spike-pseudo particle entry [190]. A broad-spectrum antiviral peptide derived from mouse beta defensin-4 shows vital antiviral functions against many viruses, including human coronaviruses [191]. Recently, it has been shown that a basic peptide derived from frog defensin inhibits the replication of the influenza virus and SARS-CoV-2 in animal models [192]. Moreover, HR2P, a peptide derived from the HR2 domain of the MERS-CoV spike, inhibits viral replication by targeting the virus–host cell membrane fusion and inhibiting cell–cell fusion [193]. By exploiting in silico and molecular biology approaches, a highly conserved motif essential for the spike-mediated fusion called “Ex3Lx6L” (E-L-L motif) was identified in the HR2 domain of coronaviruses [194]. A genetic mutation in the ELL motif or treatment with an FDA-approved drug, posaconazole, that targets the ELL motif effectively blocks the spike-mediated syncytia formation and viral infection.

## 7. FDA-Approved Inhibitors of Syncytium Formation

During the COVID-19 pandemic, researchers and scientists around the world took an urgent and collaborative approach to find potential treatments for the disease. One of the strategies used was drug repurposing, where existing FDA-approved drugs with known safety profiles were screened to identify potential therapeutics for COVID-19 [12,195,196,197]. One such drug-repurposing screening revealed that several medicines, including niclosamide, clofazimine, and salinomycin, strongly inhibit SARS-CoV-2 spike-mediated syncytia formation [12]. In the early stages of SARS-CoV-2 infection, virus-induced syncytia are beneficial for viral replication, cell-to-cell transmission, and the evasion of host defence responses. The study further revealed that Vero E6 cells become resistant to SARS-CoV-2 replication when treated with the anti-fusogenic drugs niclosamide, clofazimine, and salinomycin [12]. The effect of niclosamide on spike-induced syncytia formation is discussed in detail in the TMEM16F section.

Clofazimine, an FDA-approved essential medicine for leprosy, also possesses strong anti-mycobacterial and anti-inflammatory activity [198]. Clofazimine was initially identified as one of many anti-SARS-CoV-2 drugs in a large-scale screening conducted by Sumit K Chanda and co-workers [199]. Later, it was shown that clofazimine inhibits SARS-CoV-2 proliferation by selectively targeting the viral helicase and spike-mediated syncytia formation. The therapeutic administration of clofazimine in hamsters followed by infection with SARS-CoV-2 and MERS-CoV viruses showed a reduced viral load in the lungs and significantly lowered the viral shedding in faeces [197].

Salinomycin is an antibiotic and chemotherapeutic drug potent against many viruses, including coronaviruses [200,201]. It has been proposed that salinomycin, an ionophore, can impair viral entry by interrupting the acidification of the endosomes [202]. Salinomycin also functions as an autophagy stimulator. Autophagy induction affects SARS-CoV-2 replication [203,204,205]. Its anti-fusogenic property against the spike might be associated with the acidification of the cellular organelles, but this needs further investigation. Salinomycin has recently been identified as an anti-SARS-CoV-2 molecule in a large-scale FDA-approved screening [201]; however, the exact molecular mechanisms of how it limits the virus infection are largely elusive. In a similar study, researchers tested a series of anti-viral compounds, including inhibitors of furin protease (6-D-Arg), cathepsin B/L (E64D), lysosome acidification (concanavalin A, NH4Cl), membrane fusion (Arbidol), and hydroxychloroquine (HCQ) on spike-mediated fusion [15]. As expected, all the tested drugs effectively inhibit spike processing, and membrane fusion and cell-in-cell formation [15]. The study also suggests that spike-induced syncytia ingest lymphocytes, which may lead to cell-mediated lymphocyte death and, subsequently, contribute to lymphocytopenia (a decrease in the number of lymphocytes) in COVID-19 patients. A small-scale in silico screening of FDA-approved drugs against the ACE2 receptor identified anidulafungin and lopinavir as effective inhibitors of spike-pseudoparticles entry and syncytia formation [206]. The hydrophobic nature of anidulafungin might perturb the cell membrane lipid dynamics, which, in turn, impairs spike-mediated syncytia formation; however, the mechanism of how anidulafungin decreases spike-mediated cell fusion requires further investigation.

Berbamine hydrochloride inhibits SARS-CoV-2 infection and spike-mediated cell–cell fusion [207]. Further, molecular docking results revealed that berbamine hydrochloride potentially binds the post-fusion core of the S2 subunit with the residue S943 of the SARS-CoV-2 spike [207]. Moreover, the spike-S943 residue enhances the interaction between HR1 and HR2 to stabilise the structure of the S2 subunit and promote membrane fusion [208]. Therefore, there is a possibility that, by binding with the S943 of the S2 subunit, berbamine hydrochloride blocks the interaction between the HR1 and HR2 domains, leading to inhibitory effects on membrane fusion.

Nelfinavir inhibits syncytia formation induced by the spike of SARS-CoV and SARS-CoV-2 [209]. It is predicted that nelfinavir directly binds to the trimeric form of the spike-fusogenic domain and might inhibit cell–cell fusion [209]. In silico docking experiments showed the possibility that nelfinavir could bind to the S2 N-terminus within the S trimer and, in turn, may inhibit the formation of the heptad-repeat complex that causes spike-mediated membrane fusion.

Cobicistat is an FDA-approved pharmacokinetic enhancer, which is usually used as a booster of HIV-1 protease inhibitors (24343782). It targets the host drug-metabolising proteins, cytochrome P450-3As (CYP3As) and P-glycoprotein [210]. It has been shown that cobicistat inhibits SARS-CoV-2 replication by the inhibition of spike-mediated syncytia formation [211]. The study further demonstrates that a combination of cobicistat with remdesivir effectively reduces the SARS-CoV-2 load in Syrian hamsters [211].

Some anti-fusogenic inhibitors protect the cells from virus-induced tissue damage and slow down the virus load; thus, these drugs might be therapeutically relevant to reducing SARS-CoV-2 and VOC-associated pathologies in infected people in the future.

## 8. Neutralising Antibodies

It is well-reported that syncytia accelerate disease progression in patients infected with pathogenic viruses such as a respiratory syncytial virus [212] and HIV-1 [213,214], potentially because of extensive cell death, abnormal cytokines production, and tissue damage. A strikingly similar phenotype was noticed during the late stages of SARS-CoV-2 infection, as evident in the lung biopsies of patients who died from COVID-19 [10,215,216,217]. The syncytia formation by the fusogenic F protein of the respiratory syncytial virus is inhibited by the palivizumab and motavizumab neutralising antibodies [218]. These neutralising antibodies inhibit F protein-mediated syncytia formation by arresting the pre-fusion complexes. Many researchers across the globe identified neutralising antibodies against SARS-CoV-2, which saved the lives of many patients infected with the deadly SARS-CoV-2 [219,220,221]. The 5A6 IgG antibody inhibits spike-mediated syncytia formation in a dose-dependent manner. However, the authors also observed an opposite effect with the 3D11 antibody [222]. A better understanding of the syncytia induced by the SARS-CoV-2 spike and the mechanisms of cellular factors modulating the spike’s fusogenic properties will help to develop better COVID-19 therapeutic agents.

## 9. Conclusions and Translational Perspectives

Understanding the molecular mechanisms of syncytia formation and the modulation of spike-mediated cell fusion during virus replication is crucial in combating COVID-19. Here, we recapped the spike-syncytia formation, its involvement in virus replication, and the progression of COVID-19. As our understanding deepens, there are several exciting and essential questions that are worth exploring in COVID-19 research:

1. Since fusogenic proteins, including the spike, need to overcome barriers formed between neighbouring cells and modify cellular structures to create a competent fusion environment to begin the giant multinucleated syncytia, it would be crucial to figure out how coronavirus spike proteins manipulate cellular homeostasis and cytoskeleton to regulate the curvature formation of the host plasma membrane.
2. SARS-CoV-2 exploits both the channel and scramblase activity of the host TMEM16F to induce syncytia formation in an ACE2-dependent manner. It would be essential to figure out how ACE2-TMEM16F interacts with and contributes to the giant syncytia formation.
3. Considering that thrombocytopenia and extensive lung damage caused by SARS-CoV-2 are associated with spike-mediated syncytia formation, it is of great interest to study why and how the virus dislocates the homeostasis of cellular organelles.
4. Since cell–cell fusion inducers are important in nanocarrier biomedical research, cancer immunotherapy, and reprogramming differentiated human cells into pluripotent ones, it would be interesting to exploit the fusogenic potential of virally encoded fusion proteins, including coronavirus spike glycoproteins, for translational research.

Our understanding of how SARS-CoV-2 exploits several host factors and pathways to form syncytia is considerably increasing, substantially contributing to the comprehension of virus replication and associated pathologies. A systematic investigation is crucial and provides new insights into syncytia formation during SARS-CoV-2 infections, specifically from the perspective of cell biology and cytoskeleton remodelling. A comprehensive understanding of cellular factors during spike-mediated syncytia formation is helpful for figuring out novel approaches to effectively control infection/viral transmission and relieve SARS-CoV-2-associated pathological damages.

## Data Availability

Not applicable.

## Abbreviations

COVID-19: Coronavirus disease 2019, SARS-CoV: Severe acute respiratory syndrome coronavirus, MERS-CoV: Middle East respiratory syndrome coronavirus, ACE2: Angiotensin-converting enzyme 2, DPP4: transmembrane dipeptidyl peptidase 4, HA: hemagglutinin, gp41: glycoprotein 41, HIV-1: human immunodeficiency virus 1, VSVG: vesicular stomatitis virus glycoprotein, gB: glycoprotein B, FAST: fusion-associated small transmembrane, TM: transmembrane, ER: endoplasmic reticulum, RBD: receptor-binding domain, FP: fusion peptide, 6-HB: 6-helical bundle, HR heptapeptide repeat, VOCs: variants of concern, WHO: world health organisation, COPI: coatomer complex I, TMPRSS2: Transmembrane Serine Protease 2, ADAM: disintegrin and metalloprotease, SADS-CoV: swine acute diarrhoea syndrome coronavirus, IFITMs: Interferon-induced transmembrane proteins, ZMPSTE24: Zinc metallopeptidase STE24, CH25H: Cholesterol 25-hydroxylase, MHV-68: Murine gammaherpesvirus-68, LY6E: Lymphocyte antigen 6E, Gal-3BP: Galectin-3-binding protein, SERCA: sarcoendoplamic reticulum Ca^2+^ ATPase, HCQ: hydroxychloroquine, CYP3As: cytochrome P450-3As, CHIKV: Chikungunya virus, DNV: Dengue virus, HSV: Herpes simplex virus, BrRV: Broome orthoreovirus and RRV: Reptilian orthoreovirus.
